# Melioidosis in South America

**DOI:** 10.3390/tropicalmed3020060

**Published:** 2018-06-05

**Authors:** Dionne B. Rolim, Rachel Ximenes R. Lima, Ana Karoline C. Ribeiro, Rafael M. Colares, Leoniti D. Q. Lima, Alfonso J. Rodríguez-Morales, Franco E. Montúfar, David A. B. Dance

**Affiliations:** 1 Post-Graduation Program in Medical Sciences, University of Fortaleza (UNIFOR), Fortaleza CE 60811-905, Brazil; quel0505@gmail.com (R.X.R.L.); anakarolfreire@gmail.com (A.K.C.R.); rafaelmcolares@gmail.com (R.M.C.); leadanquei@gmail.com (L.D.Q.L.); 2 Medicine School, Ceara State University (UECE), Fortaleza CE 60714-903, Brazil; 3 Public Health and Infection Research Group, Faculty of Health Sciences, Universidad Tecnologica de Pereira, Pereira 660003, Risaralda, Colombia; ajrodriguezmmd@gmail.com; 4 Infectious Diseases Section, Pablo Tobón Uribe Hospital, Medellín 05001000, Colombia; frmontufar@yahoo.com; 5 Pulmonology Unit, León XIII Clinic of Antioquia University, Medellín 05001000, Colombia; 6 Research Group in Respiratory and Infectious Diseases (GIERI), Medellín 05001000, Colombia; 7 Lao-Oxford-Mahosot Hospital-Wellcome Trust Research Unit, Microbiology Laboratory, Mahosot Hospital, Vientiane, Laos; david.d@tropmedres.ac; 8 Centre for Tropical Medicine & Global Health, University of Oxford, Oxford OX3 7FZ, UK; 9 London School of Hygiene and Tropical Medicine, London WC1E 7HT, UK

**Keywords:** melioidosis, *Burkholderia pseudomallei*, South America

## Abstract

Melioidosis is an emerging disease in the Americas. This paper reviews confirmed cases, the presence of *Burkholderia pseudomallei* and the organization of national surveillance policies for melioidosis in South America. Confirmed cases in humans have been reported from Ecuador, Venezuela, Colombia, Brazil, and Peru. The bacterium has been isolated from the environment in Brazil and Peru. The state of Ceará, northeastern region of Brazil, is the only place where specific public strategies and policies for melioidosis have been developed. We also discuss the urgent need for health authorities in South America to pay greater attention to this disease, which has the potential to have a high impact on public health, and the importance of developing coordinated strategies amongst countries in this region.

## 1. Introduction

Melioidosis is an infectious disease caused by *Burkholderia pseudomallei*, an environmental bacterium that is being detected with increasing frequency across the world but that has only attracted attention in the Americas in the past few years. Limmathurotsakul et al., in a recent review, estimated that some 165,000 cases of the disease occurred each year in tropical areas of the globe, accounting for approximately 89,000 deaths [1]. In this study, a substantial part of South America, including northern, north-eastern and mid-western regions of Brazil, northeastern Colombia and south-western Venezuela, and parts of Peru, Guyana, Suriname, Paraguay, Bolivia and Argentina, was considered to represent a suitable environment for the bacterium [1].

Melioidosis is known to occur in the American continent; however, the true distribution of the disease is undetermined. In South America, northeastern Brazil is known to be a definite endemic area [1] and, by 2015, it had accounted for 32 of the 48 (67%) published cases from the whole continent (Table 1) [2]. This study broadens the perspective on melioidosis in South America by reviewing reported cases and exploring the organisation of public policies. Despite the relative paucity of published cases, melioidosis undoubtedly deserves to be included in the lists of infectious diseases of potential epidemiologic importance in the region. 

## 2. Review of Published Melioidosis Cases and the Presence of *B. pseudomallei*

**Figure 1:**
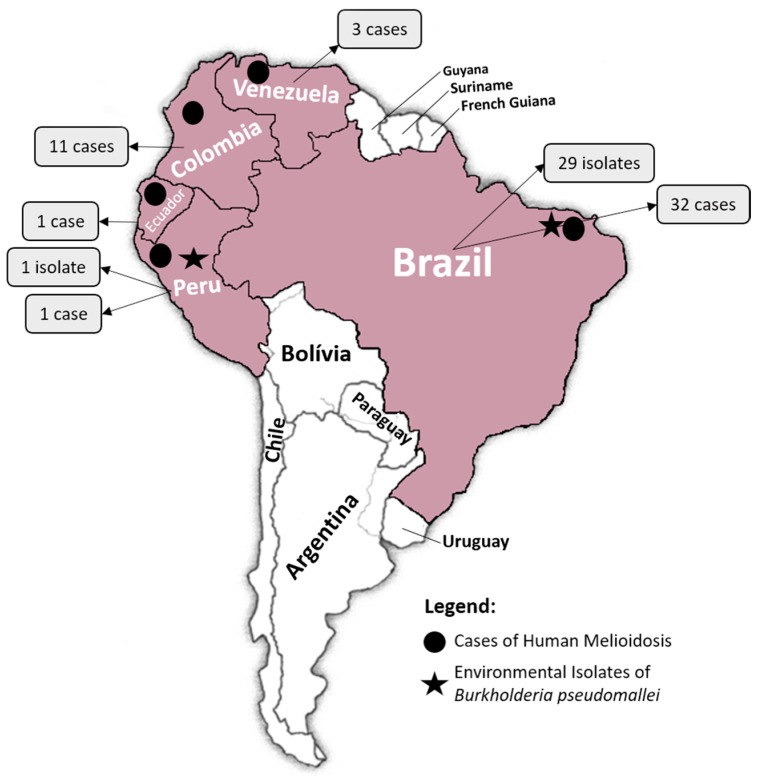
Melioidosis in South America.

In 1962, in Ecuador, Biegeleisen reported the first case of melioidosis in South America [3]. Fifteen years later, French researchers reported the isolation of *B. pseudomallei* from Brazilian and Peruvian soil [24,25]. Since these early descriptions, only sporadic human cases of melioidosis had been reported in four countries by the 1990s: Venezuela, Colombia, Brazil and Peru (in order of first reporting) (Figure 1) [2,13,21]. A genetic study, which included isolates from Ecuador, Venezuela and Brazil, suggested that isolates of *B. pseudomallei* from Central and South America probably had an African origin and were most likely imported between 1650 and 1850, as a result of colonization and the slave trade [26].

### 2.1. Venezuela, Colombia and Peru

Three cases of human melioidosis have been described in Venezuela from 1995 to 2003 [4,6]. In Colombia, after the first report in 1998 [27], a total of 11 further cases have been described up to 2015 (Table 1) [1,2,3,4,5,6,13,14,19,20,21,22,24,25,26,27,28,29]. Initially, the reports were clustered in the region of Antioquia, where there is a university referral hospital, although two cases were reported from other regions. In Peru, despite the initial report of environmental isolation, it was not until 2016 that the first human case was reported [21].

### 2.2. Brazil

**Figure 2:**
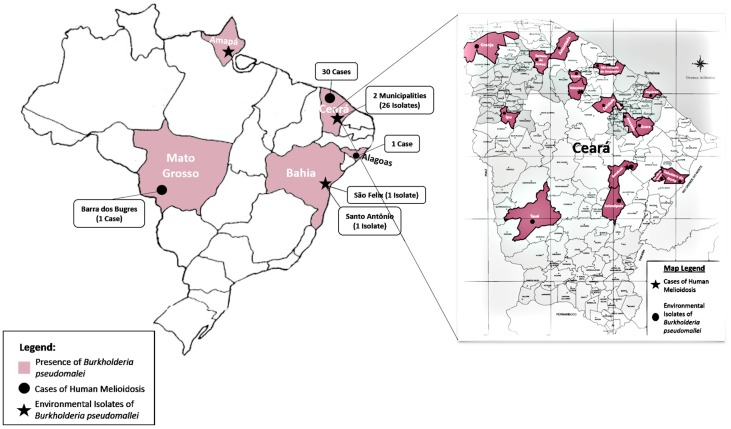
Melioidosis in Brazil.

The first documented cases of melioidosis in Brazil occurred in 2003 in the state of Ceará, located in the Northeastern region. Four siblings acquired acute infection after recreational exposure at the town dam and three of them died within a week [7,8,9,10]. By 2017, 30 cases had been diagnosed in Ceará [2,11,15,16,17,18,23]. Characterisation of clinical and environmental *B. pseudomallei* from Ceará showed that considerable genetic diversity is present [30]. Apart from this, there are only two other states with confirmed cases in Brazil: a case reported in 2007 in the state of Mato Grosso, located in the Midwestern region [12] and the other in Alagoas, also located in Northeastern region (Figure 2) [2].

### 2.3. Environmental Isolation

Besides the environmental isolation of *B. pseudomallei* reported in Peru and Brazil in 1977 [25], it has also been isolated in the Brazilian states of Ceará [31] and Amapá [32], located in the Northern region. The first attempts at environmental isolation in Brazil were made in 1973 in rice plantations in rural São Paulo, but these were unsuccessful [33].

### 2.4. Animal Melioidosis

There have been no reports of melioidosis in animals anywhere in South America.

## 3. Surveillance and Public Policies in South America

Limmathurotsakul et al. estimated the respective annual incidence and mortality of melioidosis in South American countries to be as follows: Brazil, 872 and 339; Colombia, 157 and 64; Venezuela, 103 and 40; Peru, 39 and 16; Argentina, 18 and 7; Paraguay, 13 and 5; and Bolivia, 13 and 7. [1]. These numbers are relatively small in comparison to the estimated number of cases in Asia. Nonetheless, if accurate this means that more than 1200 cases and nearly 500 deaths could be occurring annually in South America, the majority of which are going undetected and untreated. So whilst it is clearly not the top priority for public health authorities in the region, melioidosis still warrants some attention. What, then, is the position in South America as far as public health policies are concerned?

Fifteen years have passed since melioidosis was first diagnosed and documented in Brazil. There is not, however, any national recognition of it as a public health problem. Undoubtedly there are many reasons why melioidosis has not been prioritized. Other well-known and high-impact infectious diseases, such as chikungunya and Zika virus infection, have competed for attention and resources. Ceará is the only state that has developed specific policies and undertaken research into the disease. The first plan was established in 2004 following the family cluster described above, with the proposal of epidemiologic surveillance for the Secretaria de Saúde do Ceará (SESA), a government organization [34]. This document led to the publication of decree 1786/2005 establishing melioidosis as compulsorily notifiable in the state. Although there is an official national list of notifiable diseases, each federal unit has the autonomy to make its own list according to local needs. Since then, measures to enhance surveillance and management of melioidosis in Ceará have been established gradually, such as the development of a complete protocol in Brazilian Portuguese covering issues such as: definition of a suspect case, notification and environmental investigations; procedures for diagnosis and treatment, workflows for the submission of laboratory samples, and recommendations for prevention. Other activities were developed: professional training materials, dissemination of information about melioidosis at local, regional and national scientific events, research and development, the use of new media and educational technology (for example, the establishment of a web site: https://www.melioidose.com.br), trials of developing prospective surveillance in health services, and raising laboratory diagnostic capacity. However, it would be fair to say that the success of these initiatives has been limited. Professional awareness remains restricted to small groups of physicians in Fortaleza, the state capital of Ceará. In addition, the general population does not know about the disease, with rare exceptions such as in the locations where cases have been confirmed.

The scarcity and limited capabilities of microbiology laboratories in most regions of Brazil is also important in this respect. Preliminary studies in Ceará have demonstrated that few laboratories are able to identify *B. pseudomallei*. In this state, most cases have occurred in small communities and the diagnosis was only made when patients were transferred to larger reference centres located in Fortaleza or the northern and southern regions of the state. 

As far as the rest of South America is concerned, there are no policies relating to melioidosis and no formal surveillance for the disease. Even in Colombia, where the greatest number of cases outside Brazil have been detected, there are currently no specific or general public policies relating to melioidosis.Currently, in Colombia the National Institute of Health conducts indirect surveillance through the surveillance of antimicrobial resistance, but melioidosis is not statutorily notifiable and is thus likely to be under-recognised. However, a pilot study to identify cases of melioidosis in Colombia has recently been initiated. This collaborative study involving the US CDC, Tephinet, the National Institute of Health and some of the larger referral hospitals in Antioquia, will develop an epidemiological surveillance protocol and attempt to identify new cases of melioidosis, which will be confirmed using techniques such as MALDI-TOF and molecular methods.Once the collaborative project is completed, the newly identified cases and a more formal melioidosis surveillance program for Colombia will be announced. Concern over the emergence of melioidosis recently led to the first meeting focused on ‘Melioidosis in the Americas’ in Bogota in April 2018, which was attended by representatives from Brazil, Colombia, Dominican Republic, Ecuador, Haiti, Panama, Paraguay, Peru, Puerto Rico, Trinidad and Tobago, and the USA. A pamphlet on melioidosis in Spanish was developed, which could used by public health stakeholders to improve recognition of the disease in Latin America. It is hoped that this meeting will be the basis for further collaborations and perhaps even a regional network to share information to improve melioidosis surveillance.

## 4. Current Challenges and Perspective

The biggest challengesrelating to melioidosis in South America are:

1. To raise professional and government awareness that this disease is present.
2. To alert clinicians to the fact that its early detection is critical if lives are to be saved.

In Ceará, new approaches have been designed such as the inclusion of melioidosis in the curriculum of professional physicians, and the development of capacity amongst laboratory staff. The Brazilian health system is organized according to the Sistema Único de Saúde (SUS), geared to address the priorities for the local population. A recent pilot study involved the provision of information about melioidosis to community health agents in cities and towns in rural areas. These agents are part of the Family Health Strategy, a pillar of the development of primary care. Historically, these professionals have played a major role in reducing the mortality from infectious diseases in infants, such as diarrhoea, pneumonia, and measles [35]. Considering the environmental, social and geographic context of melioidosis, it is possible that using this network might help to raise the awareness of melioidosis in the wider community. Lessons learned in Brazil might then be extended to the rest of the continent. In addition, efforts should be made to integrate melioidosis into the infectious diseases agenda of pan-American meetings and organisations such as the Pan-American Health Organisation, in order to raise the profile of the disease and to initiate discussions with national public health organisations, universities and academic communities across the continent. Consideration should be given to making the disease notifiable in countries where it is predicted to be present.

An additional problem for South America is the relative under-development of the diagnostic microbiology sector. This is not something that warrants specific attention as far as melioidosis is concerned, but as laboratory capacity across the continent improves, it will be important to ensure that technicians are trained in the isolation and identification of *B. pseudomallei*. It is hoped that developments in Brazil and Colombia, and the recent meeting in Bogota, will help to catalyse the development of an integrated laboratory network among South American countries.

Although melioidosis is a relatively new disease to South America and the numbers of confirmed cases are as yet small, it is likely that it is being substantially under-diagnosed. It is only by sharing experiences about organizing and planning surveillance activities in different places, taking into consideration the individuality of each set of circumstances, that we may begin to understand the size and extent of the problem. Shared dialogue, discussion and co-ordinated actions in both Portuguese and Spanish may be the first step to reach the goal. We believe that it is essential that health authorities (national, state and municipal) should pay attention to this emerging problem. Investment in strategies already initiated must continue if the lack of knowledge about melioidosis in the continent is to be addressed.

## Figures and Tables

**Table 1:** Published Cases of Melioidosis in South America.

Case	Age	Gender	Year of Diagnosis	Country of Diagnosis	Country Where Infection Was Most Likely Acquired	Outcome	References
1	30	M	1962	Ecuador	Ecuador	Died	[3]
2	50	M	1995	Venezuela	Venezuela	Survived	[4]
3	60	M	1998	Colombia	Colombia	Survived	[5]
4	65	M	1998	Colombia	Colombia	Survived	[5]
5	50	M	2000	Venezuela	Venezuela	Survived	[4]
6	66	M	2003	Portugal	Venezuela	Survived	[6]
7	10	F	2003	Colombia	Colombia	Died	[5]
8	40	M	2003	Colombia	Colombia	Died	[5]
9	15	M	2003	Brazil	Brazil	Died	[7,8,9,10]
10	14	F	2003	Brazil	Brazil	Died	[7,8,9,10]
11	10	M	2003	Brazil	Brazil	Died	[7,8,9,10]
12	12	F	2003	Brazil	Brazil	Survived	[7,8,9,10]
13	50	M	2003	Netherlands	Brazil	Died	[11]
14	46	F	2004	Colombia	Colombia	Survived	[5]
15	28	M	2005	Spain	Colombia	Survived	[5]
16	52	M	2005	Colombia	Colombia	Survived	[5]
17	17	F	2005	Brazil	Brazil	Survived	[12]
18	30	M	2005	Brazil	Brazil	Died	[2,13]
19	22	M	2008	Colombia	Colombia	Survived	[14]
20	17	M	2008	Brazil	Brazil	Died	[15]
21	69	M	2008	Brazil	Brazil	Died	[16,17]
22	48	M	2009	Brazil	Brazil	Survived	[17]
23	47	M	2010	Brazil	Brazil	Survived	[17]
24	28	M	2010	Brazil	Brazil	Died	[18]
25	29	M	2010	Brazil	Brazil	Died	[2]
26	56	M	2010	Brazil	Brazil	Survived	[2]
27	53	M	2011	Brazil	Brazil	Survived	[2]
28	3	M	2011	Brazil	Brazil	Died	[2]
29	56	M	2011	Brazil	Brazil	Died	[2]
30	7	M	2011	Brazil	Brazil	Survived	[2]
31	29	M	2011	Brazil	Brazil	Survived	[2]
32	21	M	2012	Brazil	Brazil	Died	[2]
33	82	F	2012	Brazil	Brazil	Died	[2]
34	31	M	2012	Colombia	Colombia	Survived	[5]
35	36	M	2013	Colombia	Colombia	Survived	[19,20]
36	19	F	2016	Peru	Peru	Died	[21]
37	68	M	2013	Brazil	Brazil	Died	[2]
38	57	M	2014	Brazil	Brazil	Died	[2]
39	42	M	2014	Brazil	Brazil	Survived	[2]
40	57	M	2014	Brazil	Brazil	Survived	[2]
41	50	M	2014	Brazil	Brazil	Died	[2]
41	72	M	2014?	Colombia	Colombia	Survived	[22]
42	42	M	2015	Brazil	Brazil	Survived	[2]
43	13	F	2015	Brazil	Brazil	Died	[23]
44	64	M	2016	Brazil	Brazil	Survived	[23]
45	58	M	2016	Brazil	Brazil	Died	[23]
46	54	M	2016	Brazil	Brazil	Survived	[23]
47	100	M	2017	Brazil	Brazil	Died	[23]
48	4	F	2017	Brazil	Brazil	Survived	[23]
